# In This Issue: Volume 116, Issue 3, March 2025

**DOI:** 10.1111/cas.70031

**Published:** 2025-03-03

## Mechanisms of resistance to KRAS inhibitors: cancer cells' strategic use of normal cellular mechanisms to adapt

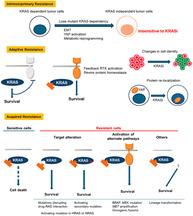

KRAS is a protein that regulates cell growth, but when mutated, it can drive cancer development. These mutations were commonly found in lung, pancreatic, and colorectal cancers, making KRAS a crucial target for cancer therapy. For years, scientists struggled to inhibit KRAS because it lacked obvious drug‐binding pockets. However, the discovery of the switch‐II pocket led to the development of specific KRAS inhibitors, including sotorasib and adagrasib, offering new therapeutic options for patients with previously untreatable cancers.

In this review, Tanaka and Ebi reviewed the mechanisms of resistance to KRAS inhibitors and strategies to overcome them. Tumors with KRAS gain‐of‐function mutations initially depend on the mutant KRAS for growth but may develop intrinsic resistance by activating alternative pathways. This resistance results from mechanisms such as epithelial‐to‐mesenchymal transition (EMT), YAP activation, or KEAP1 mutations. Additionally, cancer cells rewire their signaling pathways to bypass KRAS inhibition. For example, feedback reactivation of MAPK signaling, triggered by receptor tyrosine kinases, supports tumor survival. KRAS inhibition also disrupts protein homeostasis, but cells adapt by reactivating MAPK or AKT pathways.

The researchers highlighted how cancer cells undergo metabolic reprogramming and protein re‐localization to counteract KRAS inhibition. The re‐localization of E‐cadherin and Scribble from the membrane to the cytosol causes YAP to move to the nucleus, where it promotes MRAS transcription, leading to MAPK reactivation. Additionally, changes in cell identity, such as lineage switching from adenocarcinoma to squamous cell carcinoma, contribute to the drug resistance.

Acquired resistance to KRAS inhibitors also occurs through secondary mutations in KRAS or alterations in related signaling proteins. Mutations in the switch‐II pocket hinder drug binding, while other oncogenic mutations reduce drug effectiveness. To address these challenges, researchers are developing next‐generation KRAS inhibitors, broad‐spectrum drugs, and combination therapies targeting multiple pathways. Immunotherapy has been also explored as a complementary approach.

This review provided valuable insights into the complex mechanisms of resistance to KRAS inhibitors and highlighted innovative therapeutic strategies aimed at improving patient outcomes.

https://onlinelibrary.wiley.com/doi/10.1111/cas.16441

## Tumor exosomal RNPEP promotes lung metastasis of liver cancer via inducing cancer‐associated fibroblast activation

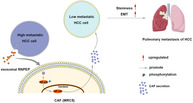

Hepatocellular carcinoma (HCC), or liver cancer, is the fourth leading cause of cancer‐related death in the world. One reason for HCC's high mortality is its high tendency to metastasize and spread to other organs, such as the lungs. In fact, metastasis in the lungs is particularly significant as it accounts for many HCC‐related deaths.

Exosomes are nanoparticles secreted by cells that play a crucial role in HCC metastasis. Normally, they act as couriers, carrying molecular cargo (e.g., proteins, lipids, and nucleic acids) from one cell to another. Exosomes secreted by HCC cells can transform fibroblasts—cells that maintain connective tissues—into cancer‐associated fibroblasts (CAFs). CAFs are known to modulate the tumor microenvironment (TME), a complex ecosystem that surrounds a tumor, and facilitate metastasis. This makes CAFs a potential target for curbing HCC metastasis. However, the mechanism through which exosomes activate CAFs need to be understood.

RNPEP (arginyl aminopeptidase) is an extracellular enzyme involved in the development of many malignancies. Bioinformatics analysis revealed that RNPEP is overexpressed in HCC cells. This study focused on the role of RNPEP in exosomes‐induced CAF activation.

The researchers found that RNPEP was highly expressed (found in abundance) in the plasma of patients with HCC. Moreover, exosomes secreted from metastatic HCC cells showed overexpression of RNPEP. When exposed to the human fibroblast cell line MRC‐5, the exosomes induced CAF‐like properties in the fibroblasts such as expressing CAF biomarkers and increased cellular mobility. Once reprogrammed by RNPEP‐carrying exosomes, the MRC‐5 fibroblasts improved the stemness—the ability of a cell to continuously self‐renew—of metastatic HCC cells (MHCC‐97 L). Reprogrammed MRC‐5 cells also promoted epithelial–mesenchymal transition (EMT) of MHCC‐97 L cells, a process where cells transition into a stem cell‐like state.

But how does RNPEP reprogram fibroblasts? The researchers found that RNPEP activates NF‐κB signaling in MRC‐5 fibroblasts, which is a critical pathway associated with CAF activation.

RNPEP knockdown—reducing the gene's expression—in the exosomes secreted by metastatic HCC cells reduced the metastatic intensity of the cancer. The researchers also demonstrated the pro‐metastatic effects of RNPEP in vivo, by transplanting (xenografting) exosome‐reprogrammed MRC‐5 into mice.

This study shows that exosome carrying RNPEP, secreted by HCC cells, can reprogram fibroblasts into CAFs via NF‐κB signaling pathway. This crosstalk between RNPEP and CAF promotes HCC metastasis, especially in the lungs.

https://onlinelibrary.wiley.com/doi/10.1111/cas.16417

## TIM‐3 marks measurable residual leukemic stem cells responsible for relapse after allogeneic stem cell transplantation

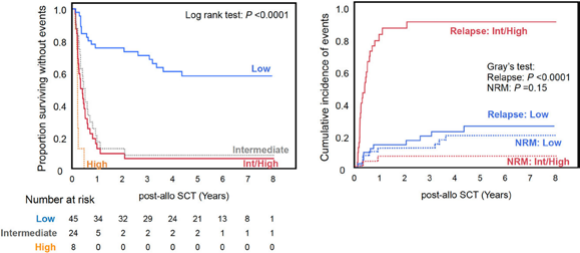

Relapse following allogeneic stem cell transplantation (allo‐SCT) remains a significant challenge for patients with acute myeloid leukemia (AML). Despite advancements in treatment, residual leukemic stem cells (LSCs)—which are resistant to chemotherapy—can persist in the bone marrow and contribute to disease recurrence.

This study identifies T‐cell immunoglobulin mucin‐3 (TIM‐3) as a crucial marker for predicting relapse risk in AML. Researchers analyzed bone marrow samples from AML patients after allo‐SCT and found that TIM‐3 was exclusively expressed on leukemic stem cells but absent from healthy donor‐derived stem cells.

The findings indicate that:

- Patients with a high TIM‐3^+^ LSC burden (≥60%) experienced a significantly higher relapse rate (87.5%) and shorter survival.
- Patients with a low TIM‐3^+^ LSC burden (<60%) had a lower relapse risk (22.2%) and improved long‐term survival.

These results suggest that early assessment of TIM‐3^+^ LSCs post‐transplantation may serve as a valuable prognostic tool. Identifying patients with a high risk of relapse could allow for closer monitoring, early therapeutic interventions, or targeted strategies to eliminate residual LSCs.

Furthermore, the study highlights the potential for TIM‐3‐targeted therapies, such as antibodies, to reduce relapse risk and improve long‐term outcomes. By incorporating TIM‐3 as a predictive marker, clinicians may be able to refine post‐transplant management strategies, ultimately enhancing survival and quality of life for AML patients.

https://onlinelibrary.wiley.com/doi/epdf/10.1111/cas.16431

